# Automatic Detection of Epilepsy and Seizure Using Multiclass Sparse Extreme Learning Machine Classification

**DOI:** 10.1155/2017/6849360

**Published:** 2017-06-19

**Authors:** Yuanfa Wang, Zunchao Li, Lichen Feng, Chuang Zheng, Wenhao Zhang

**Affiliations:** ^1^School of Electronic and Information Engineering, Xi'an Jiaotong University, Xi'an 710049, China; ^2^Guangdong Xi'an Jiaotong University Academy, Shunde 528300, China

## Abstract

An automatic detection system for distinguishing normal, ictal, and interictal electroencephalogram (EEG) signals is of great help in clinical practice. This paper presents a three-class classification system based on discrete wavelet transform (DWT) and the nonlinear sparse extreme learning machine (SELM) for epilepsy and epileptic seizure detection. Three-level lifting DWT using Daubechies order 4 wavelet is introduced to decompose EEG signals into delta, theta, alpha, and beta subbands. Considering classification accuracy and computational complexity, the maximum and standard deviation values of each subband are computed to create an eight-dimensional feature vector. After comparing five multiclass SELM strategies, the one-against-one strategy with the highest accuracy is chosen for the three-class classification system. The performance of the designed three-class classification system is tested with publicly available epilepsy dataset. The results show that the system achieves high enough classification accuracy by combining the SELM and DWT and reduces training and testing time by decreasing computational complexity and feature dimension. With excellent classification performance and low computation complexity, this three-class classification system can be utilized for practical epileptic EEG detection, and it offers great potentials for portable automatic epilepsy and seizure detection system in the future hardware implementation.

## 1. Introduction

Epilepsy is one of the most common chronic neurological disorders and is a condition with recurrent evoking of seizure. Nowadays, about one percent of population in the world is suffering from epilepsy [1], which costs billions of dollars annually for direct medical care. Epileptic seizure impacts the quality of life for patients and their families and even leads to the death of patients. Therefore, detecting and curing epilepsy with high efficiency are very necessary. Electroencephalogram (EEG), which shows the temporal and spatial information of brains' electrical voltages, is successfully used to diagnose epilepsy patients [2]. Currently, the seizure detection relies on “interviewing” patients and inspecting EEG recordings by highly trained professionals in hospitals [3, 4]. However, this approach is extremely inaccurate and inconvenient, and epilepsy patients may show normal states when their seizures do not occur. Differentiating between healthy and interictal (seizure-free) EEG signals can be used to diagnose epilepsy in a clinical setting and, additionally, the detection of seizure is of importance for instant treatment [5]. So, automatic classification of healthy, ictal (seizure), and interictal EEG signals is of great clinical significance.

The machine learning approach is generally used to the automatic detection of seizure EEG signals. Many machine learning methods have been used for EEG classification [6–15]. Artificial neural network (ANN) has been widely applied to classify EEG signals over the last two decades [8]. However, the conventional learning algorithms for ANN, such as the backpropagation (BP) algorithm, are prone to fall into a local minimum [9]. It is very time-consuming to adjust the connection weights and biases in ANN, and the learning speed is too slow to meet the requirements of practical applications, which has been a major bottleneck for development [9]. Another popular machine learning method, support vector machine (SVM), has been successfully used to classify epileptic EEG signals [10–12]. However, since the training of SVM involves a quadratic programming (QP) problem, the computational complexity of SVM training algorithms is usually intensive, which is at least quadratic with respect to the number of training examples. So it is difficult to deal with large problems using SVM [10]. Extreme learning machine (ELM) is an emerging machine learning method which was proposed by Huang et al. [13, 14] for the generalized single hidden layer feedforward neural networks (SLFN), in which the hidden node parameters are randomly generated and the output weights are analytically computed [13, 14]. ELM is successfully applied to detect seizure EEG in previous works [7, 9]. However, the initial ELM would consume large storage space if implemented in hardware in calculating the inverse of matrix [15, 16]. The sparse ELM (SELM), state-of-the-art algorithm, was proposed in [16]. Similar to the conventional SVM, the training of SELM is essentially a QP problem. The only difference between them is that the SELM does not have the sum constraint [16]. In the SELM, as fewer constraints need to be satisfied, and only one Lagrange variable needs to be updated in each iteration, the training process would be easier. Consequently, compared with SVM and ANN, the SELM needs less storage space and takes shorter training and testing time. With all the advantages, the SELM provides a more efficient way for hardware implementation and satisfies the demand for portable seizure detection application.

The SELM was originally proposed for binary classification. Different strategies based on SVM have been proposed for multiclass classification problems [17–20], such as one-against-all (OAA), one-against-one (OAO), binary tree (BT), error-correcting output codes (ECOC), and directed acyclic graph SVM (DAG-SVM). Inspired by the multiclass SVM, the binary SELM classifier can also be extended for multiclass classification by constructing and combining several binary classifiers together. For achieving good performance in the three-class classification of epileptic EEG signals, nonlinear SELM classifiers with different kernel functions are compared and kernel parameters are optimized simultaneously in this work. Eventually, we find that OAO strategy with Gaussian SELM is the best multiclass classification for epilepsy and seizure detection.

The feature extraction of EEG signals plays an important role in the performance of multiclass classification [21]. The methods of feature extraction used can be categorized into four types: time domain, frequency domain, time-frequency domain, and nonlinear analysis [21, 22]. For nonstationary EEG signals, discrete wavelet transform (DWT) has been proved to be an efficient tool due to its ability to resolve the signals in both time and frequency domains [2]. The DWT filters are conventionally designed based on the convolution operation architecture [23] which requires many complex operations and large memory [24]. To overcome these drawbacks, the lifting-based DWT (LDWT) is adopted and implemented. There are many types of wavelet transforms such as Haar, Mexican Hat, Gaussian, Morlet, and Daubechies wavelets, of which Daubechies 4 (db4) wavelet is found to be the most appropriate for epileptic EEG analysis because its wave characteristic is similar to the spike wave of the EEG signals [25].

Selection of the SELM input is important since even the best classifier will perform poorly if the input is not selected well [22, 26]. Although some previous feature selection methods can increase the detection performance [27, 28], they suffer from the high dimensionality of features, and the complexity makes hardware implementation difficult and expensive. In this work, three-level LDWT is used to decompose the EEG signals into delta, theta, alpha, and beta subbands; the feature values of each subband are computed to create multidimensional feature vectors. In order to obtain maximum accuracy with a low-dimensional feature vector under certain conditions, a great number of combinations of different features are investigated. Finally, the maximum and standard deviation values of each subband are calculated to create eight-dimensional feature vectors as the input to the multiclass SELM classification.

To the best of our knowledge, this work is the first work to design a three-class classification system based on LDWT and the multiclass SELM for detecting epilepsy and seizure. This paper makes two contributions. First of all, this paper develops a low computational complexity for feature extraction and multiclass classification that can detect epilepsy and seizure with high enough classification accuracy. What is more, this paper provides a good solution for portable automatic epilepsy and seizure detection system.

The rest of this paper is organized as follows.  Section 2 describes the methods of the multiclass classification system, including the SELM algorithm, multiclass classification strategy, and feature extraction based on LDWT.  Section 3 describes the experimental results and discussions of the proposed epilepsy and seizure detection system.  Section 4 concludes the paper.

## 2. Methods

**Figure 1:**
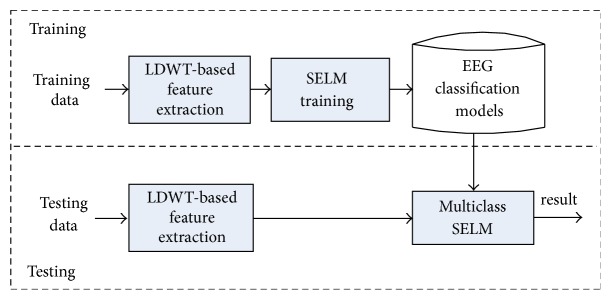
Workflow of the proposed EEG classification system.

This section will present the multiclass classification system for epilepsy and seizure detection. The multiclass classification system consists of two phases: training and testing phases.  Figure 1 shows the workflow of the proposed EEG classification system. EEG signals are decomposed into one approximation and three detailed coefficients using the three-level LDWT, and then eight features are extracted by computing the maximum and standard deviation values of the wavelet coefficients (discussed in detail in what follows). The eight-dimensional feature vectors are input to the multiclass SELM. Labelled EEG signals are used for training the system, and, after training, unlabelled EEG signals can be automatically classified into normal, interictal, or ictal ones by the multiclass SELM system.

In this section, we first review the SELM algorithm and present the five strategies of the multiclass SELM and then introduce the LDWT-based feature extraction.

### 2.1. Binary SELM

Training binary SELM in classification is also equivalent to solving the inequality constrained convex QP problem which can be written as follows [14]:

(1) Minimize: Ld=12∑i=1N ∑j=1Nαiαjtitjkxi,xj−∑i=1NαiSubject  to: 0≤αi≤C,i=1,…,N,

where* N* is the number of training samples, *α*_*i*_ is the Lagrange multiplier, *t*_*i*_ ∈ {±1} is the associated class label, and* C* is a predefined regularization constant. *k*(*x*_*i*_, *x*_*j*_) is kernel function that is used for nonlinear classification, and the kernel functions could be, but not limited to, the following [16]. 

Gaussian kernel is

(2) $$\begin{array}{c} k\left(\;x_{i},x_{j}\;\right)=\exp\left(\;-\frac{{\left\|x_{i}-x_{j}\right\|}^{2}}{2\sigma^{2}}\;\right). \end{array}$$

Laplacian kernel is

(3) $$\begin{array}{c} k\left(\;x_{i},x_{j}\;\right)=\exp\left(\;-\frac{{\left\|x_{i}-x_{j}\right\|}^{2}}{\sigma }\;\right),\;\sigma >0. \end{array}$$

Polynomial kernel is

(4) $$\begin{array}{c} k\left(\;x_{i},x_{j}\;\right)={\left(\;1+x_{i}\cdot x_{j}\;\right)}^{m},\;m\in Z^{+}. \end{array}$$

The training algorithm of the SELM is summarized as follows.

Since only one Lagrange multiplier needs to be updated in each iteration [16], choosing the updated *α*_*c*_ is vital. The index *c* of the updated *α*_*c*_ in each iteration is determined according to

(5) $$\begin{array}{c} c=arg\underset{i=1,\ldots ,N}{\min}\;J_{i}, \end{array}$$

where *J*_*i*_ = *g*_*i*_ · *d*_*i*_, *g*_*i*_ = (∂/∂*α*_*i*_)*L*_*d*_ denotes the gradient of *L*_*d*_, and *d*_*i*_ indicates the way in which *α*_*i*_ should be updated, expressed as follows:

(6) $$\begin{array}{c} d_{i}=\left\{\begin{array}{cc} 1, & \alpha_{i}=0\\ -sign\left(g_{i}\right), & 0<\alpha_{i}<C\\ -1, & \alpha_{i}=C. \end{array}\right. \end{array}$$

The corresponding Lagrange variable *α*_*c*_ is updated as follows:

(7) $$\begin{array}{c} \alpha_{c}^{new}=\alpha_{c}^{old}-g_{c}^{old}. \end{array}$$

The unconstrained point must be checked to ensure that it is in the feasible range [0, *C*], and the clipped function can be written as follows:

(8) $$\begin{array}{c} \alpha_{c}^{new,clip}=\left\{\begin{array}{cc} 0, & \alpha_{c}^{new}<0\\ \alpha_{c}^{new}, & \alpha_{c}^{new}\in \left[0,C\right]\\ C, & \alpha_{c}^{new}>C. \end{array}\right. \end{array}$$

After updating *α*_*c*_, *g*_*i*_  (*i* = 1,2,…, *N*) is updated as follows:

(9) $$\begin{array}{c} g_{i}^{new}=g_{i}^{old}+t_{i}\cdot t_{c}\cdot k\left(\;x_{i},x_{c}\;\right)\cdot \left(\;\alpha_{c}^{new}-\alpha_{c}^{old}\;\right). \end{array}$$

Based on the updated values of *g*_*i*_ and *d*_*i*_, *J*_*i*_'s (*i* = 1,2,…, *N*) are updated according to the definition.

Repeat the above iteration until min_*i*=1,…,*N*_ ⁡*J*_*i*_ > −*ε* is satisfied [16], where *ε* is a preset tolerance.

When the training stage is finished and the SELM parameters are determined, we can classify a new object *x* with

(10) $$\begin{array}{c} f\left(\;x\;\right)=sign\left(\;\overset{N_{s}}{\underset{i=1}{\sum }}\alpha_{i}t_{i}k\left(\;x,x_{i}\;\right)\;\right), \end{array}$$

where *N*_*s*_ is the number of nonzero Lagrange multipliers. The pseudocode of the SELM training algorithm is summarized in Algorithm 1.

### 2.2. Multiclass SELM Strategy

Even though the SELM is designed for binary classification, it can be extended for multiclass classification by constructing and combining several binary classifiers together. In the multiclass SELM, we discuss the five typical strategies, namely, OAA, OAO, BT, ECOC, and DAG [17–20, 36]. For the three-class problem (normal, interictal, and ictal EEG signals), we make a brief introduction of these approaches.

(*1) OAA* (see [17]). Here three binary SELM classifiers are trained, in which the* i*th SELM is trained with all of the samples in the* i*th class with positive (+1) labels and all the other samples with negative (−1) labels. After training, three decision functions ∑_*i*=1_^*N*^*α*_*i*_^1^*t*_*i*_^1^*k*(*x*, *x*_*i*_), ∑_*i*=1_^*N*^*α*_*i*_^2^*t*_*i*_^2^*k*(*x*, *x*_*i*_), and ∑_*i*=1_^*N*^*α*_*i*_^3^*t*_*i*_^3^*k*(*x*, *x*_*i*_) are used to determine the class of an unknown sample* x* as follows:

(11) $$\begin{array}{c} \text{class of }x\equiv \arg\underset{j=1,2,3}{\max}\left(\;\overset{N}{\underset{i=1}{\sum }}{\alpha_{i}}^{j}{t_{i}}^{j}k\left(\;x,x_{i}\;\right)\;\right). \end{array}$$

(*2) OAO* (see [17]). Here three binary SELM classifiers are trained, and each classifier is trained using samples from a pair of classes. After training, three decision functions sign(∑_*i*=1_^*N*^*α*_*i*_^12^*t*_*i*_^12^*k*(*x*, *x*_*i*_)), sign(∑_*i*=1_^*N*^*α*_*i*_^23^*t*_*i*_^23^*k*(*x*, *x*_*i*_)), and sign(∑_*i*=1_^*N*^*α*_*i*_^13^*t*_*i*_^13^*k*(*x*, *x*_*i*_)) are used to determine the class of an unknown sample *x* by the majority vote strategy. In the vote strategy, if sign(∑_*i*=1_^*N*^*α*_*i*_^*pq*^*t*_*i*_^*pq*^*k*(*x*, *x*_*i*_)) says that *x* is in the* p*th class, then the vote for the* p*th class is added by one; otherwise the* q*th is increased by one; then we predict that *x* is in the class with the largest vote. However, if each class has the same vote number, we say *x* is in the class which has the largest absolute function value. For example, if |∑_*i*=1_^*N*^*α*_*i*_^12^*t*_*i*_^12^*k*(*x*, *x*_*i*_)| is the largest one in the three functions, the final class is determined by the decision function sign(∑_*i*=1_^*N*^*α*_*i*_^12^*t*_*i*_^12^*k*(*x*, *x*_*i*_)).

(*3) DAG* (see [17]). Its training phase is the same as the OAO strategy, and three binary SELM classifiers are trained. DAG depends on a rooted binary directed acyclic graph to make a decision. When an unknown sample *x* reaches the leaf node, the final decision will be made.

(*4) ECOC* (see [18]). Its training phase is the same as the OAA strategy. One SELM classifies class 1 from classes 2 and 3, a second SELM classifies 2 from 1 and 3, and a third SELM classifies 3 from 1 and 2. Samples from classes 1, 2, and 3 have target codes (1, −1, −1), (−1,1, −1), and (−1, −1,1), respectively. Given an unknown sample, the three SELM classifiers should be used to determine the actual output code. The sample is assigned to the class with the closest target code in the Hamming distance sense.

(*5) BT* (see [19, 20]). For three classes (1, 2, and 3), we need two classifiers. For example, the first SELM classifies 3 from 1 and 2, and the second SELM classifies 1 from 2. When an unknown sample is fed into the BT, class 3 is fully separated by the first classifier, and class 1 and class 2 can be classified by the second classifier.

### 2.3. LDWT-Based Feature Extraction

**Figure 2:**
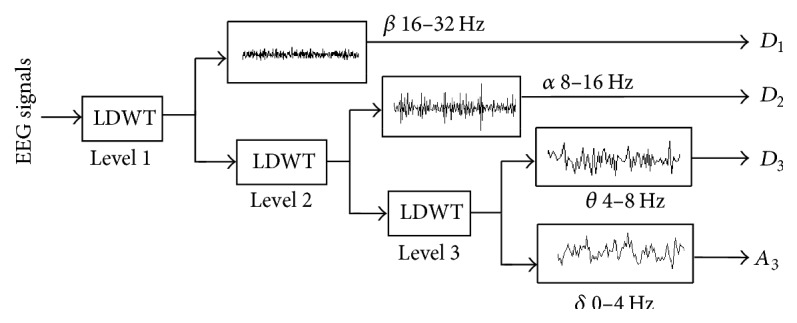
Three-level wavelet decomposition structure.

Figure 2 shows the three-level wavelet decomposition structure. Because the main frequencies of epileptic EEG signals are below 32 Hz [2], they are preprocessed by a band-pass filter between 0 Hz and 32 Hz. The three-level LDWT decomposes each EEG signal into four subbands, generating the approximation coefficient *A*_3_ with the frequency range of 0–4 Hz corresponding to the delta wave and detail coefficients *D*_1_ with the frequency range of 16–32 Hz corresponding to the beta wave, *D*_2_ with the frequency range of 8–16 Hz corresponding to the alpha wave, and *D*_3_ with the frequency range of 4–8 Hz corresponding to the theta wave.

The *z*-domain transfer functions *G*(*z*) and *H*(*z*) of the low-pass and high-pass db4 filters are as follows [37]:

(12) Gz=h0+h1z−1+h2z−2+h3z−3,Hz=−h3+h2z−1−h1z−2+h0z−3,

where $h(0)=\left(1+\sqrt{3}\right)/4\sqrt{2}$, $h(1)=\left(3+\sqrt{3}\right)/4\sqrt{2}$, $h(2)=\left(3-\sqrt{3}\right)/4\sqrt{2}$, and $h(3)=\left(1-\sqrt{3}\right)/4\sqrt{2}$.

Using lifting scheme [37], the polyphase matrix of db4 wavelet can be factored into lifting steps as follows:

(13) $$\begin{array}{c} \tilde{P}\left(\;z\;\right)=\left[\begin{array}{cc} 1 & -\sqrt{3}\\ 0 & 1 \end{array}\right]\left[\begin{array}{cc} 1 & 0\\ \frac{\sqrt{3}}{4}+\frac{\sqrt{3}-2}{4}z^{-1} & 1 \end{array}\right]\left[\begin{array}{cc} 1 & z\\ 0 & 1 \end{array}\right]\left[\begin{array}{cc} \frac{\sqrt{3}+1}{\sqrt{2}} & 0\\ 0 & \frac{\sqrt{3}-1}{\sqrt{2}} \end{array}\right]. \end{array}$$

After decomposing the EEG signals into four coefficients, the feature values of the wavelet coefficients are computed to create multidimensional feature vectors. In this work, the maximum, minimum, mean, variance, approximate entropy, sample entropy, autocorrelation, and standard deviation values are extracted as features and input to the three-class classification. However, using all of the features may not improve the classification accuracy but cause high complexity if implemented in hardware design. One objective of this paper aims at reducing the computational complexity while maintaining certain classification accuracy. In order to obtain maximum accuracy with a low-dimensional feature vector under certain conditions, classification accuracy is calculated with 4 different feature dimensions, that is, 4, 8, 12, and 16. For each feature dimension, a great number of combinations of different features are investigated. Eventually eight-dimensional feature vectors by computing maximum and standard deviation values of the wavelet coefficients are fed into the three-class SELM classification.

## 3. Experimental Results and Discussions

In this section, the EEG datasets are summarized, and the performance of three-class classification is evaluated. The experiment and simulation are conducted with MATLAB R2010a on a 3.30 GHz Intel(R) Core(TM) i5-4590 processor with 4 GB memory.

### 3.1. EEG Data

The EEG dataset from the University of Bonn, Germany, is used to test the performance [38]. The dataset contains 5 subsets (A–E), which are recorded intracranially on humans for a presurgical evaluation of focal epilepsies, each with 100 single-channel EEG segments. A summary of the 5 subsets is given in Table 1.

Since subsets A, D, and E are used in most of the epilepsy and seizure detection methods [29–31], these subsets are also selected to evaluate the proposed three-class classification system. Subset A contains surface EEG recordings from five healthy volunteers with their eyes open, subset D includes intracranial EEG recordings of five patients during seizure-free intervals from within the epileptogenic zone of the brain, and subset E is recorded during the seizures of five epileptic volunteers. The sample frequency of the EEG dataset is 173.61 Hz, and each segment has 4096 points.

In data preprocessing, every segment is divided into 512-point sliding time epochs with 256-point overlap between adjacent epochs, the length of each epoch is 2.94 s, and there is an overlap of 1.47 s between adjacent epochs [10]. Overall, 1600 epochs are constructed from each subset for a total of 4800 epochs over the three subsets A, D, and E. Fourfold cross-validation is used to evaluate the performance of the proposed system. In 4-fold cross-validation, 4800 epochs are partitioned into 4 mutually exclusive parts of approximately equal size, and each part is called fold. In each time, one fold is used for testing and the remaining three folds are put together for training. Then the average performance across all trails is calculated.

### 3.2. Performance Evaluation

The performance of the proposed multiclass SELM system can be evaluated by sensitivity, specificity, and total classification accuracy, which are defined as follows [10, 39]:

(14) Sensitivity=number  of  true  positive  decisionsnumber  of  actually  positive  cases.Specificity=number  of  true  negative  decisionsnumber  of  actually  negative  cases.Total  classification  accuracy=number  of  correct  decisionstotal  number  of  cases.

Table 2 presents the confusion matrix of the three-class SELM. S_AD_ represents the sum of epochs from set D and is classified by the proposed system as epochs from set A, and the other parameters can be interpreted similarly.  Table 3 shows the detailed definition of the three-class classification measures.

### 3.3. Comparative Study and Results

**Figure 3:**
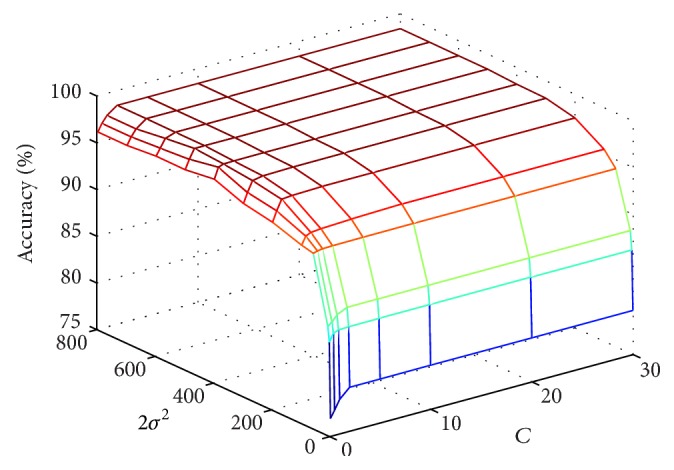
Accuracy of Gaussian SELM with different values of *C* and 2*σ*^2^.

**Figure 4:**
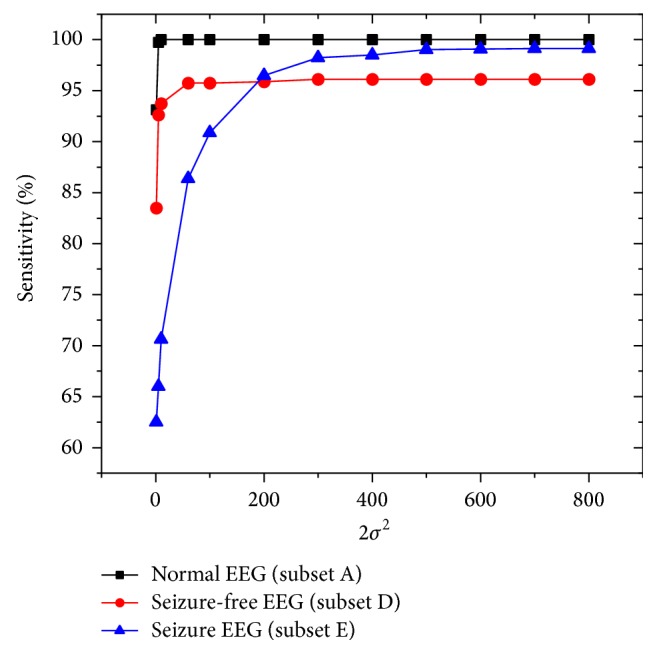
Sensitivities using the three subsets versus 2*σ*^2^.

In order to achieve a good performance, the tolerance *ε* and the parameters of kernel function need to be chosen appropriately. First, we select the tolerance *ε* to be 0.001, which can ensure enough high accuracy [16]. In this work, Gaussian kernel and polynomial kernel are selected since they achieve better generalization performance for most applications [16]. Before comparing the five multiclass classifications, parameter combination of cost parameter* C* and kernel parameter *σ*^2^ or *m* should be chosen a priori. Taking OAO, for example, the following method is used to find the appropriate parameters* C* and *σ*^2^ of Gaussian SELM. The cost parameter* C* and kernel parameter *σ*^2^ have different influence on the classification performance of the Gaussian SELM. 2*σ*^2^ is tuned with 12 different values, that is, 1, 5, 10, 60, 100, 200, 300, 400, 500, 600, 700, and 800, and* C* is tuned with 8 different values, that is, 0.1, 0.5, 1, 2, 5, 10, 20, and 30. Using the three subsets, the accuracy of Gaussian SELM with different values of C and 2*σ*^2^ is shown in Figure 3. Our experiments demonstrate that the classification performance of the Gaussian SELM is not very sensitive to the parameter* C* within a certain range. We select *C* = 5 for the OAO strategy in this work. However, 2*σ*^2^ has rather great effect on the epilepsy and seizure detection. In order to further examine this classification effect and determine the appropriate value of 2*σ*^2^, the sensitivities using the three subsets 2*σ*^2^ are shown in Figure 4 when* C* takes 5. As can be seen from Figure 4, the sensitivities using the three subsets all tend to be constant when 2*σ*^2^ = 500. But they will decrease if 2*σ*^2^ is too large, which is not displayed in Figure 4. So 2*σ*^2^ is set to 500 in OAO strategy.

For polynomial SELM, parameter* m* is tried with 7 different values, that is, 1, 2, 3, 4, 5, 10, and 20, and* C* is also tuned with 8 different values mentioned before. Using the same method, the appropriate values of parameters* C* and* m* of polynomial SELM can be obtained.

We only need to determine the appropriate parameter values for OAO and OAA strategies since the other strategies only differ in combining methods. The used parameter values of* C*, 2*σ*^2^, and* m* in the five strategies are shown in Table 4.

**Figure 5:**
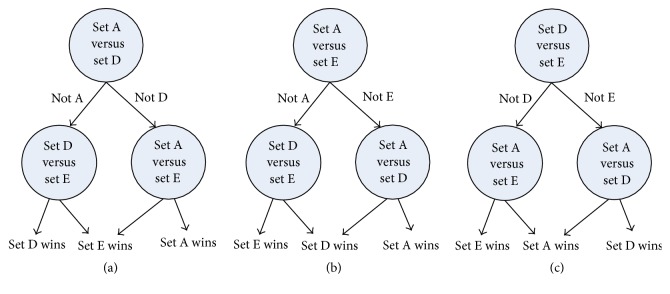
Three DAG structures generated for the three-class problem.

**Figure 6:**
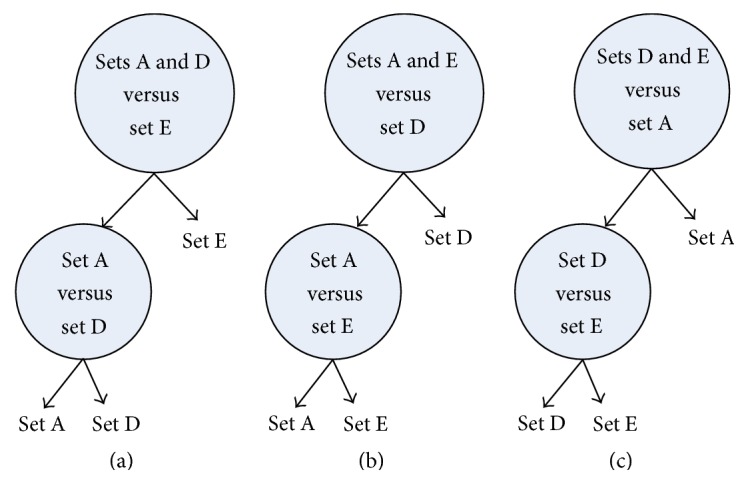
Three BT structures generated for the three-class problem.

In order to find out the most efficient three-class classification strategy, the classification sensitivities of the five mentioned strategies are compared. Moreover, it should be noted that three structures can be used in three-class classification problem in DAG and BT strategies. Figures 5 and 6 show the DAG and BT structures, respectively. In this work, the three-class SELM yields the classification accuracy of 96.9%, 97.2%, and 97.1% using the structures in Figures 5(a), 5(b), and 5(c), respectively, while those values are 96.7%, 96.1%, and 96.6% using the structures in Figures 6(a), 6(b), and 6(c), respectively. Therefore, the structures in Figures 5(b) and 6(a) are selected to compare with other strategies. Tables 5 and 6 show the sensitivities of all the five strategies using Gaussian SELM and polynomial SELM, respectively. It can be found that OAO strategy with Gaussian SELM achieves the highest sensitivity. Therefore, OAO strategy with Gaussian SELM is chosen to study the performance of the multiclass classification in what follows.

Once the OAO strategy with Gaussian SELM has been selected, specificity and total classification accuracy are also calculated to further evaluate the three-class classification. The sensitivity, specificity, and total classification accuracy are given in Table 7. In order to compare the performance between Gaussian SELM and Gaussian SVM, LIBSVM is used for training and testing the Gaussian SVM.  Table 8 shows the comparison of Gaussian SELM and Gaussian SVM in classification accuracy, training time, and testing time with the same features and sample data. As can be seen from Table 8, the training and testing time spent by SELM is much shorter than that spent by SVM.

### 3.4. Comparison and Discussion

In order to further explore the significance of the proposed three-class classification, this section provides the comparisons of our approach with other reported methods and discusses the results of the comparisons.  Table 9 summarizes the performance comparison of this work with previous works including binary classification and multiclass classification for epileptic EEG detection. As can be seen from Table 9, a combination of DWT and ELM has been used for binary classification between ictal and interictal EEG signals [22]. However, the feature extraction and classifier in [22] have high complexity if implemented in hardware. This work is the first work to implement the multiclass SELM based on LDWT for epilepsy and seizure detection. As observed from Table 9, this work has the highest accuracy for three-class classification except the one in [30], but it requires easier training process and less storage space than the latter. In addition, its feature extraction has the lowest computational complexity in all the systems in Table 9. Therefore the proposed three-class classification can be successfully used in hardware implementation for portable automatic epilepsy and seizure detection system.

In order to compare this work with [32, 33], the five subsets (A–E) are classified into three categories. The EEG signals from sets A and B are labelled as the healthy class, the signals from sets C and D are grouped as the interictal class, and the signals from set E are labelled as the seizure class. The performance of the proposed method is also verified using the three categories of EEG signals. The sensitivities of the signals from subsets (A, B), (C, D), and E are 98.1%, 96.3%, and 98.4%, respectively.  Table 10 summarizes the accuracy comparison between the proposed method and [32, 33] using subsets (A, B), (C, D), and E. As we know, ANN used in [32, 33] requires a more extensive training process and complicated design procedure. As shown in Table 10, the proposed system achieves higher accuracy than [32] and similar accuracy to [33] but uses fewer features than [33].

Although the Bonn datasets have been used by many studies to test their EEG analysis algorithms, they have some limitations, one of which is that the Bonn datasets about epilepsy patients are obtained by using intracranial electrodes [27]. Considering that the intracranial recordings is not always available in the clinic [27, 32], the open CHB-MIT scalp EEG database [40] is also used to verify the effectiveness of classification algorithms in some studies. As is known, the CHB-MIT scalp EEG database is collected from epilepsy patients and therefore only includes the seizure class and the seizure-free class. But there is no open scalp EEG database which includes the above three classes and gets widely used. Considering that the proposed three-class classifier is composed of binary classifiers, the CHB-MIT database is also chosen to verify the effectiveness of the SELM for scalp EEG signals, in which Channels FP1–F7 covering the frontal region of the brain are selected.  Table 11 summarizes the comparison between the binary SELM classifier and the existing literature using the CHB-MIT scalp EEG database. As can be seen from Table 11, the sensitivity and specificity of the binary SELM classifier are 81.1% and 98.3%, respectively. The classifiers in [34, 35] require more extensive training and complicated design than SELM if implemented in hardware.

From the experiments and discussions, with the advantages of high enough classification performance, low complexity, and easy training process, the proposed three-class classification system exhibits excellent practical value especially in the future hardware implementation for portable automatic epilepsy and seizure detection system.

## 4. Conclusion

Automatic EEG detection system is of great significance for epilepsy diagnosis. A three-class classification system based on LDWT and the SELM is designed to detect epilepsy and seizure for the first time. A lifting-based db4 wavelet transform is introduced to speed up the computation of feature extraction. After optimizing the parameters of Gaussian kernel and polynomial kernel, the performances of the five multiclass SELM strategies are compared, and the majority voting-based OAO strategy with Gaussian SELM is chosen for the three-class classification because of its highest accuracy. The three-class classification system is tested using the publicly available epilepsy dataset including normal, seizure activity, and seizure-free EEG signals. Simulation results show that the designed system achieves high enough classification accuracy by combining LDWT and the SELM. In addition, this system reduces training and testing time by decreasing computational complexity and feature dimension. It is a valuable system for future hardware implementation of automatic multiclass EEG classification.

## Figures and Tables

**Algorithm 1:**
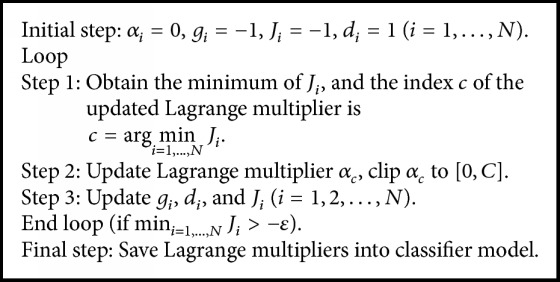
Pseudocode of the binary SELM algorithm.

**Table 1:** Summary of the clinical data.

	Set A	Set B	Set C	Set D	Set E
Patient state	Awake and eyes open (normal)	Awake and eyes closed (normal)	Seizure-free (interictal)	Seizure-free (interictal)	Seizure activity (ictal)
Electrode types	Surface	Surface	Intracranial	Intracranial	Intracranial
Electrode placement	International 10–20 systems	International 10–20 systems	Opposite to epileptogenic zone	Within epileptogenic zone	Within epileptogenic zone
Number of segments	100	100	100	100	100
Segment duration (s)	23.6	23.6	23.6	23.6	23.6

**Table 2:** Confusion matrix.

Output/desired	Set A	Set D	Set E
Set A	S_AA_	S_AD_	S_AE_
Set D	S_DA_	S_DD_	S_DE_
Set E	S_EA_	S_ED_	S_EE_

**Table 3:** Performance definition.

	Sensitivity (%)	Specificity (%)	Accuracy (%)
Set A	$\frac{S_{AA}}{S_{A}}$	$\frac{\left(S_{DD}+S_{DE}+S_{ED}+S_{EE}\right)}{\left(S_{D}+S_{E}\right)}$	$\frac{\left(S_{AA}+S_{DD}+S_{EE}\right)}{total}$
Set D	$\frac{S_{DD}}{S_{D}}$	$\frac{\left(S_{AA}+S_{AE}+S_{EA}+S_{EE}\right)}{\left(S_{A}+S_{E}\right)}$	
Set E	$\frac{S_{EE}}{S_{E}}$	$\frac{\left(S_{AA}+S_{AD}+S_{DA}+S_{DD}\right)}{\left(S_{A}+S_{D}\right)}$	

**Table 4:** Parameters of Gaussian kernel and polynomial kernel.

Strategy	Gaussian kernel		Polynomial kernel	
	*C*	2*σ*^2^	*C*	*m*
BT	5	500	10	4
OAO	5	500	10	4
OAA	5	600	10	3
DAG	5	500	10	4
ECOC	5	600	10	3

**Table 5:** Sensitivities of various multiclass classification strategies using Gaussian SELM.

	BT (%)	OAO (%)	OAA (%)	DAG (%)	ECOC (%)
Set A	98.8	100	98.0	98.8	96.0
Set D	93.8	96.3	94.5	94.9	93.7
Set E	97.5	99.0	97.5	98.0	96.2

**Table 6:** Sensitivities of various multiclass classification strategies using polynomial SELM.

	BT (%)	OAO (%)	OAA (%)	DAG (%)	ECOC (%)
Set A	97.5	99.2	97.5	98.5	96.0
Set D	93.2	96.0	94.0	95.0	92.9
Set E	99.5	98.0	98.6	97.5	96.1

**Table 7:** Results of the OAO three-class classification system using subsets A, D, and E.

	Sensitivity (%)	Specificity (%)	Accuracy (%)
Set A	100	99.0	98.4
Set D	96.3	99.6	
Set E	99.0	99.0	

**Table 8:** Comparison of SELM and SVM.

Classification method	Accuracy (%)	Training time (s)	Testing time (s)
SVM	96.8	1.315	0.051
SELM	98.4	0.592	0.032

**Table 9:** Comparison with previous works.

Authors (year)	Classifier	Feature extraction	Classes	Subsets	Accuracy (%)
Tang and Durand [10] (2012)	SVM	Filter bank, Teager energy, power, Lempel–Ziv complexity	2	(A, D), E	98.72
Song et al. [22] (2016)	Initial ELM	DWT, Mahalanobis distance, sample entropy	2	D, E	97.53
Güler et al. [29] (2005)	ANN	Lyapunov exponents	3	A, D, E	96.79
Liang et al. [30] (2010)	SVM	Principal component analysis, approximate entropy, power	3	A, D, E	98.67
Murugavel and Ramakrishnan [8] (2016)	SVM	DWT, largest Lyapunov exponent, approximate entropy	3	A, D, E	96
Riaz et al. [31] (2016)	SVM	Empirical mode decomposition, temporal, spectral features	3	A, D, E	85
This work	SELM	LDWT, maximum, standard deviation	3	A, D, E	98.4

**Table 10:** Comparison with previous works using subsets (A, B), (C, D), and E.

Authors (year)	Methods	Number of features	Accuracy (%)
Alam and Bhuiyan [32] (2013)	EMD, higher order moments, and ANN	3	80
Tzallas et al. [33] (2007)	Fraction energy and ANN	40	97.72
This work	LDWT, maximum, standard deviation, and SELM	8	97.6

**Table 11:** Comparison with previous works using CHB-MIT scalp EEG.

Authors (year)	Methods	Sensitivity (%)	Specificity (%)
Samiee et al. [34] (2015)	DWT, 2D mapping and textural features, and SVM	70.19	97.74
Samiee et al. [35] (2016)	Sparse RDSTFT and LGBP, Logistic regression, random forest, and SVM	70.4	99.1
This work	LDWT, maximum, standard deviation, and SELM	81.1	98.3

## References

[1] Yoo J., Yan L., El-Damak D., Altaf M. A. B., Shoeb A. H., Chandrakasan A. P. (2013). An 8-channel scalable EEG acquisition SoC with patient-specific seizure classification and recording processor. *IEEE Journal of Solid-State Circuits*.

[2] Adelia H., Zhoub Z., Dadmehrc N. (2003). Analysis of EEG records in an epileptic patient using wavelet transform. *Journal of Neuroscience Methods*.

[3] Chen W.-M., Chiueh H., Chen T.-J. (2014). A fully integrated 8-channel closed-loop neural-prosthetic cmos soc for real-time epileptic seizure control. *IEEE Journal of Solid-State Circuits*.

[4] Bin Altaf M. A., Yoo J. (2016). A 1.83 *μ*j/classification, 8-channel, patient-specific epileptic seizure classification soc using a non-linear support vector machine. *IEEE Transactions on Biomedical Circuits and Systems*.

[5] Ghosh-Dastidar S., Adeli H., Dadmehr N. (2008). Principal component analysis-enhanced cosine radial basis function neural network for robust epilepsy and seizure detection. *IEEE Transactions on Biomedical Engineering*.

[6] Srinivasan V., Eswaran C., Sriraam N. (2007). Approximate entropy-based epileptic EEG detection using artificial neural networks. *IEEE Transactions on Information Technology in Biomedicine*.

[7] Song Y., Crowcroft J., Zhang J. (2012). Automatic epileptic seizure detection in EEGs based on optimized sample entropy and extreme learning machine. *Journal of Neuroscience Methods*.

[8] Murugavel A. S. M., Ramakrishnan S. (2016). Hierarchical multi-class SVM with ELM kernel for epileptic EEG signal classification. *Medical and Biological Engineering and Computing*.

[9] Yuan Q., Zhou W., Li S., Cai D. (2011). Epileptic EEG classification based on extreme learning machine and nonlinear features. *Epilepsy Research*.

[10] Tang Y., Durand D. M. (2012). A tunable support vector machine assembly classifier for epileptic seizure detection. *Expert Systems with Applications*.

[11] Liu Y., Zhou W., Yuan Q., Chen S. (2012). Automatic seizure detection using wavelet transform and SVM in long-term intracranial EEG. *IEEE Transactions on Neural Systems and Rehabilitation Engineering*.

[12] Altaf M. A. B., Tillak J., Kifle Y., Yoo J. A 1.83 *μ*J/classification nonlinear support-vector-machine-based patient-specific seizure classification SoC.

[13] Huang G., Chen L., Siew C. (2006). Universal approximation using incremental constructive feedforward networks with random hidden nodes. *IEEE Transactions on Neural Networks*.

[14] Huang G. B., Zhu Q. Y., Siew C. K. (2006). Extreme learning machine: theory and applications. *Neurocomputing*.

[15] Decherchi S., Gastaldo P., Leoncini A., Zunino R. (2012). Efficient digital implementation of extreme learning machines for classification. *IEEE Transactions on Circuits and Systems II: Express Briefs*.

[16] Bai Z., Huang G. B., Wang D., Wang H., Westover M. B. (2014). Sparse extreme learning machine for classification. *IEEE Transactions on Cybernetics*.

[17] Hsu C. W., Lin C. J. (2002). A comparison of methods for multiclass support vector machines. *IEEE Transactions on Neural Networks*.

[18] Güler I., Übeyli E. D. (2007). Multiclass support vector machines for EEG-signals classification. *IEEE Transactions on Information Technology in Biomedicine*.

[19] Mathur A., Foody G. M. (2008). Multiclass and binary SVM classification: implications for training and classification users. *IEEE Geoscience and Remote Sensing Letters*.

[20] Fei B., Liu J. (2006). Binary tree of SVM: a new fast multiclass training and classification algorithm. *IEEE Transactions on Neural Networks*.

[21] Acharya U. R., Sree S. V., Swapna G., Martis R. J., Suri J. S. (2013). Automated EEG analysis of epilepsy: a review. *Knowledge-Based Systems*.

[22] Song J.-L., Hu W., Zhang R. (2016). Automated detection of epileptic EEGs using a novel fusion feature and extreme learning machine. *Neurocomputing*.

[23] Hsia C.-H., Chiang J.-S., Guo J.-M. (2013). Memory-efficient hardware architecture of 2-D dual-mode lifting-based discrete wavelet transform. *IEEE Transactions on Circuits and Systems for Video Technology*.

[24] Song J., Park I.-C. (2009). Pipelined discrete wavelet transform architecture scanning dual lines. *IEEE Transactions on Circuits and Systems II: Express Briefs*.

[25] Yonga G. C., Maan N., Ahmad T. (2013). EEG signal of epiliptic patient by fast Fourier and wavelet transforms. *Jurnal Teknologi (Sciences & Engineering)*.

[26] Bajaj V., Pachori R. B. (2012). Classification of seizure and nonseizure EEG signals using empirical mode decomposition. *IEEE Transactions on Information Technology in Biomedicine*.

[27] Shen C.-P., Chen C.-C., Hsieh S.-L. (2013). High-performance seizure detection system using a wavelet-approximate entropy-fSVM cascade with clinical validation. *Clinical EEG and Neuroscience*.

[28] Siuly S., Li Y. (2015). Designing a robust feature extraction method based on optimum allocation and principal component analysis for epileptic EEG signal classification. *Computer Methods and Programs in Biomedicine*.

[29] Güler N. F., Übeyli E. D., Güler I. (2005). Recurrent neural networks employing Lyapunov exponents for EEG signals classification. *Expert Systems with Applications*.

[30] Liang S.-F., Wang H.-C., Chang W.-L. (2010). Combination of EEG complexity and spectral analysis for epilepsy diagnosis and seizure detection. *Eurasip Journal on Advances in Signal Processing*.

[31] Riaz F., Hassan A., Rehman S., Niazi I. K., Dremstrup K. (2016). EMD-based temporal and spectral features for the classification of EEG signals using supervised learning. *IEEE Transactions on Neural Systems and Rehabilitation Engineering*.

[32] Alam S. M. S., Bhuiyan M. I. H. (2013). Detection of seizure and epilepsy using higher order statistics in the EMD domain. *IEEE Journal of Biomedical and Health Informatics*.

[33] Tzallas A. T., Tsipouras M. G., Fotiadis D. I. (2007). Automatic seizure detection based on time-frequency analysis and artificial neural networks. *Computational Intelligence and Neuroscience*.

[34] Samiee K., Kiranyaz S., Gabbouj M., Saramäki T. (2015). Long-term epileptic EEG classification via 2D mapping and textural features. *Expert Systems with Applications*.

[35] Samiee K., Kovács P., Gabbouj M. (2016). Epileptic seizure detection in long-term EEG records using sparse rational decomposition and local Gabor binary patterns feature extraction. *Knowledge-Based Systems*.

[36] Huang G.-B., Zhou H., Ding X., Zhang R. (2012). Extreme learning machine for regression and multiclass classification. *IEEE Transactions on Systems, Man, and Cybernetics B: Cybernetics*.

[37] Kayhan S., Erçelebi E. (2011). ECG denoising on bivariate shrinkage function exploiting interscale dependency of wavelet coefficients. *Turkish Journal of Electrical Engineering and Computer Sciences*.

[38] Andrzejak R. G., Lehnertz K., Mormann F., Rieke C., David P., Elger C. E. (2001). Indications of nonlinear deterministic and finite-dimensional structures in time series of brain electrical activity: dependence on recording region and brain state. *Physical Review E*.

[39] Güler I., Übeyli E. D. (2005). Adaptive neuro-fuzzy inference system for classification of EEG signals using wavelet coefficients. *Journal of Neuroscience Methods*.

[40] Goldberger A. L., Amaral L. A., Glass L. (2000). PhysioBank, PhysioToolkit, and PhysioNet: components of a new research resource for complex physiologic signals. *Circulation*.

